# Characterizing HLA-A2-restricted CD8^+^ T-cell epitopes and immune responses to Omicron variants in SARS-CoV-2-inactivated vaccine recipients

**DOI:** 10.3389/fimmu.2025.1534530

**Published:** 2025-03-18

**Authors:** Chanchan Xiao, Jian Xiang, Haoyun Wang, Wen Gao, Tianchan Peng, Shumin Li, Jun Su, Xi Chen, Lijuan Gao, Ruohu Shi, Xinyi Mou, Jun Yuan, Guobing Chen

**Affiliations:** ^1^ Science and Education Department, The First Affiliated Hospital of Jinan University and The Sixth Affiliated Hospital of Jinan University, Guangzhou, Guangdong, China; ^2^ Department of Microbiology and Immunology, School of Medicine, Institute of Geriatric Immunology, School of Medicine, Jinan University, Guangzhou, China; ^3^ Key Laboratory of Viral Pathogenesis and Infection Prevention and Control (Jinan University), Ministry of Education, Guangzhou, China; ^4^ Guangdong-Hong Kong-Macau Great Bay Area Geroscience Joint Laboratory, School of Medicine, Jinan University, Guangzhou, China; ^5^ Zhuhai Institute of Jinan University, Jinan University, Zhuhai, China; ^6^ NHC Key Laboratory of Male Reproduction and Genetics, Guangdong Provincial Reproductive Science Institute (Guangdong Provincial Fertility Hospital), Guangzhou, China; ^7^ First Affiliated Hospital, Jinan University, Guangzhou, China; ^8^ Infectious Disease Prevention and Control Department, Guangzhou Centers for Disease Control and Prevention, Guangzhou, Guangdong, China

**Keywords:** SARS-CoV-2, Omicron, cellular immune response, CD8 T-cell epitope, conserved epitope

## Abstract

**Introduction:**

Recent surveillance has identified the emergence of the SARS-CoV-2 Omicron ariant, which exhibits the ability to evade multiple neutralizing antibodies generated by prior infection or vaccination. However, significant knowledge gaps remain regarding the CD8 T-cell immune reactivity to the Omicron variant. This study aims to evaluate the characteristics of HLA-A2-restricted CD8 T-cell epitopes from the Omicron variant and analyze epitope-specific CD8 T-cell responses to SARS-CoV-2 inactivated vaccines.

**Methods:**

We conducted a comprehensive analysis of CD8 T-cell responses to SARS-CoV-2 inactivated vaccines, focusing on HLA-A2-restricted epitopes derived from the Omicron variant. Mutant epitopes were evaluated for their impact on antigen presentation and CD8 T-cell immune reactivity. Additionally, we screened for epitopes that exhibited reduced CD8 T-cell responses following the emergence of the Omicron variant.

**Results:**

Our findings revealed that mutant epitopes in the Omicron variant led to escape from antigen presentation and diminished CD8 T-cell immune responses. We identified two epitopes associated with decreased CD8 T-cell reactivity post-Omicron variant emergence. Notably, we discovered an S protein epitope, 67A>V, which demonstrated similar proportions of CD8 T-cell specificity between the ancestral and mutant strains, suggesting its conservation and potential immunogenicity for vaccine development. Furthermore, the third dose of the inactivated vaccine significantly increased the number of epitope-specific CD8 T cells, underscoring the importance of booster doses in enhancing cellular immune responses against the Omicron variant.

**Discussion:**

This study highlights the ability of the Omicron variant to evade CD8 T-cell immune responses through epitope mutations, while also identifying conserved epitopes with potential utility in vaccine design. The observed increase in epitope-specific CD8 T cells following a booster dose emphasizes the critical role of additional vaccinations in strengthening cellular immunity against emerging SARS-CoV-2 variants. These findings provide valuable insights for the development of next-generation vaccines targeting conserved epitopes and optimizing booster strategies.

## Introduction

With its continuous pandemic wave, COVID-19 poses a very high health threat. According to the latest COVID-19 epidemic report from the World Health Organization, from August 19 to September 15, 2024, compared with the previous 28-day period (July 22 to August 18, 2024), the number of new COVID-19 cases and deaths worldwide increased by 10% and 11%, respectively. A total of 890,000 new cases have been reported across 89 countries, and approximately 5,700 new deaths have been reported in 31 countries (1). Therefore, the study of severe acute respiratory syndrome coronavirus 2 (SARS-CoV-2) is highly necessary. The Omicron variant, as one of the mutations of concern, is characterized by having the highest number of mutations among SARS-CoV-2 variants, with 50 mutations in its genome, including 30 mutations in the spike (S) protein. These mutations in the S protein are particularly concerning because they confer increased transmissibility and partial resistance to immunity induced by existing COVID-19 vaccines and antibody therapies. Therefore, understanding the mutations in Omicron variants is essential for developing effective countermeasures against COVID-19 (2). A recent report based on an artificial intelligence (AI) model (TopNetmAb) analyzed the influence of 15 RBD mutations on the OMIC and efficacy of existing vaccines in a simulation study. The analysis revealed that mutations at the N440K, T478K, and N501Y sites of Omicron may increase its infectivity 10-fold and 2-fold greater than those of the original SARS-CoV-2 and Delta variants, respectively (3). To confirm the analysis, an Omicron S protein (pseudotyped) construct showed an ED_50_ of 66 when tested in human sera obtained from convalescent patients with COVID-19, representing an 8.4-fold reduction in neutralization capability (4). *In vitro* studies by Wilhelm et al. revealed that the neutralization ability of Omicron was reduced by 11.4- and 20-fold in sera from double BNT162b2 vaccine recipients and double mRNA1273 vaccine recipients, respectively (5). Preliminary data suggest that this variant has an increased risk of reinfection and limited neutralizing capacity mediated by antibodies.

Variants of concern (VOCs), including Alpha (B.1.1.7), Beta (B.1.351), Gamma (P.1), Iota (B.1.526.1), Delta (B.1.617.2), and Omicron, exhibit most of their mutations in the S protein of the virus, likely due to selective evasion of the antibody response. Cao et al. reported that the Omicron lineage can evolve mutations to escape the humoral immunity caused by Omicron BA.1 infection, whereas BA.1-derived vaccine enhancers may not generate broad-spectrum protection against new Omicron variants (6). Those who were infected with an Omicron variant mainly recalled neutralizing antibodies against the original strain or other VOC variant strains and S1-specific T-cell responses. In addition, immunity to Omicron itself was very weak after vaccination with 3 doses of the BNT162b2 or CoronaVac vaccine (7). Thus, a history of infection with SARS-CoV-2 and mutations within Omicron may lead to a lack of neutralizing antibody response to infection with Omicron BA.1.

Although neutralizing antibodies are believed to be the main means by which the body resists pathogens, previous studies have suggested that, rather than neutralizing antibodies, cellular immunity plays an important role in the body’s immune defense against SARS-CoV-2. Some of the protective effects of vaccination are attributed to T-cell immunity, as CD8^+^ T cells play a key role in reducing the severity of COVID-19 and inducing long-term immune protection (8). The prevalence of detectable SARS-CoV-2-specific CD4^+^ and CD8^+^ T cells in convalescent COVID-19 patients is 100% and 70%, respectively (9). A recent report explored whether different vaccine platforms (mRNA-1273, BNT162b2, Ad26.COV2. S, and NVX-CoV2373) induced cross-reactive T-cell responses to early SARS-CoV-2 variants; the results revealed that the median number of spike epitopes recognized by CD4 and CD8^+^ T cells was 11 and 10, respectively, with average preservation > 80% for Omicron. The functional preservation of the majority of T-cell responses may play an important role in second-level defenses against diverse variants (10). Other recent data have suggested that SARS-CoV-2 spike-specific CD8 T cells induced by prior infection or BNT162b2 vaccination provide extensive immune coverage against Omicron. The median relative frequencies of SARS-CoV-2 spike-specific CD8 T cells were 70% and 92%, respectively (11). In addition, our previous study revealed that a given CD8 T-cell epitope mutation resulted in a loss of antigen presentation and impaired antigen-specific T-cell function, suggesting that viral evolution led to immune evasion (12, 13). However, whether there is a highly conserved S protein target between Omicron mutants and ancestral strains that can be used as an ideal vaccine target to induce a high frequency of T-cell immune responses is currently unknown.

In this study, we predicted potential CD8 T-cell epitopes within the ancestral strain of SARS-CoV-2 and studied those within which mutations, including MHC-I binding affinity of peptides and CD8 T-cell activation effects, occurred in the Omicron strain. We detected epitope-specific CD8 T cells in SARS-CoV-2-inactivated vaccine recipients via the corresponding tetramers. The results revealed that the Δ3674-76 mutation in ORF1a 3673-84 in the Omicron variant and the Δ69-70 mutation in S 60-71 led to decreased CD8 T-cell activation and possible immune escape. However, the number of epitope-specific CD8 T cells was significantly greater in the third-dose vaccinated subjects than in the second-dose vaccinated subjects. This finding demonstrated that after two doses of inactivated vaccines as “priming” shots, a booster dose of a third homologous inactivated vaccine improved cellular immune protection against Omicron. In addition, we identified a highly conserved S protein target between Omicron mutation strains and ancestral strains that can be used as an ideal vaccine target to induce a high frequency of T-cell immune responses, which may be effective in preventing infection by SARS-CoV-2 Omicron variants.

## Results

### Activation of T cells stimulated with T-cell epitopes containing Omicron mutations

**Figure 1:**
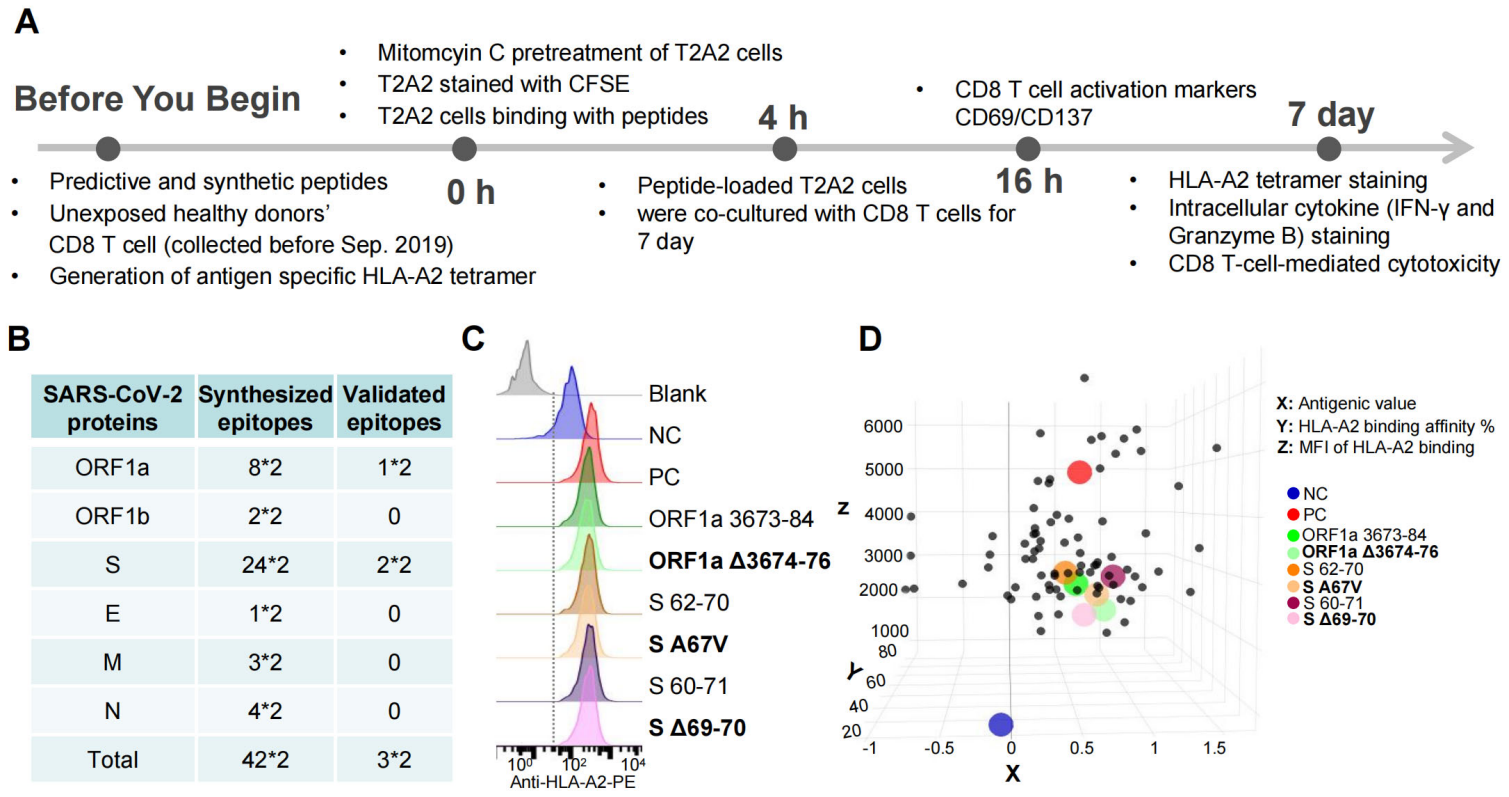
Identification of HLA-A2-restricted T-cell epitopes from the Omicron variant strain. **(A)** Timeline for the overall experimental procedures. Before antigen epitope screening and identification, we first predicted the epitopes via the IEDB database, followed by the synthesis of epitope peptides. Since the experiment required human CD8^+^ T cells, we recruited volunteers and collected peripheral blood samples. To label antigen-specific CD8^+^ T cells, we prepared tetramer antibodies in advance. At the 0-hour stage of the experiment, we used antigen-presenting T2A2 cells. As T2A2 cells need to be cocultured with CD8^+^ T cells for 7 days, we treated them with mitomycin to inhibit their proliferation. Simultaneously, we stained CFSE with T2A2 cells for fluorescent labeling, which also allowed us to monitor the inhibitory effect of mitomycin. At the 4-hour stage, we coincubated the peptides with T2A2 cells for 4 hours, followed by the addition of CD8^+^ T cells at a 1:1 ratio for coculture. At the 16-hour mark of the coculture, we detected the expression of CD69 and CD137. After 7 days of coculture, we used tetramers to label antigen-specific CD8^+^ T cells and measured the expression of IFN-γ and Granzyme B while also verifying the cytotoxic effects of activated CD8^+^ T cells on target cells. Through this series of experiments, we ultimately obtained immunogenic epitopes. **(B)** Summary of the synthesized peptides from the ancestral Wuhan-Hu-1 and Omicron strains. A total of 42 pairs of epitopes were identified, with multiplication by two indicating the inclusion of both ancestral and mutated epitopes. These epitopes are distributed across various viral proteins: ORF1a, ORF1b, S (spike), E (envelope), M (membrane), and N (nucleocapsid). Through experimental validation, as depicted in **(A)**, three pairs of epitopes were confirmed to be immunogenic. These immunogenic epitopes are located on the ORF1a and S proteins. **(C)** Comparison of the binding affinities of Omicron ancestral and mutant epitopes to HLA-A2 on antigen-presenting T2 cells. The ancestral and mutant epitopes are listed in black and bold, respectively. Paired ancestral and mutant epitopes are listed adjacent to each other. **(D)** The antigenic value is shown on the X-axis, and ancestral and mutant SARS-CoV-2 epitopes that bind to HLA-A2 via ELISA are shown on the Y-axis, whereas the Omicron ancestral and mutant epitopes that bind to HLA-A2 on antigen-presenting T2 cells are shown on the Z-axis. n=3 per experiment. Blank means no peptides; NC means negative control, EBV virus peptide IVTDFSVIK; PC means positive control, influenza A M1 peptide GILGFVFTL. Each dot represents a single individual.

**Figure 2:**
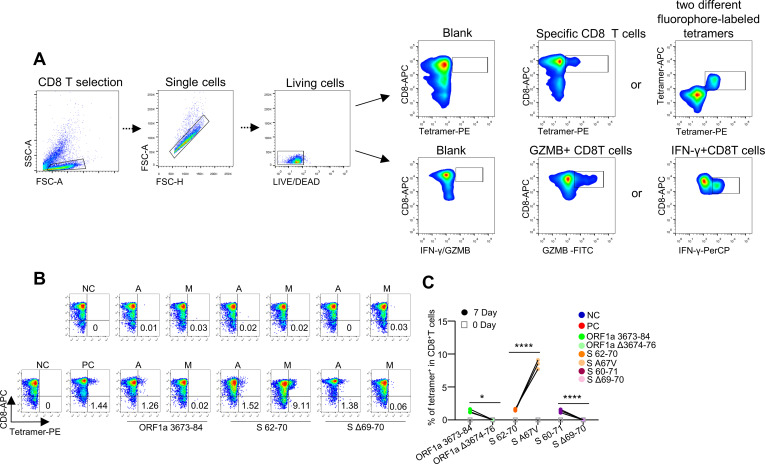
Identification of HLA-A2-restricted T-cell epitopes and activation of CD8^+^ T cells by epitopes from SARS-CoV-2. **(A)** Flow cytometry gating strategy for SARS-CoV-2 epitope-specific CD8^+^ T, GZMB^+^ CD8^+^ T, and IFNγ^+^ CD8^+^ T cells. In the coculture of T2A2 cells and CD8^+^ T cells, CD8^+^ T cells can be distinguished from T2A2 cells because they are not stained with CFSE. Therefore, we first gated CD8^+^ T cells, then selected singlets to remove cell aggregates, and finally analyzed viable CD8^+^ T cells. **(B)** Representative FACS plots of specific CD8 T cells recognized by tetramers containing the Omicron SARS-CoV-2 candidate epitope peptide. CD8^+^ T cells sorted from PBMCs from healthy donors were cocultured with antigen-presenting T2 cells loaded with various peptides for activation. The CD8 T cells were stained with the corresponding tetramer containing the ancestral or mutated epitope and compared before (day 0; top row) and after (day 7; bottom row) stimulation. Paired ancestral and mutant epitopes are placed adjacent to each other. The flow cytometry gating strategy is shown in **(A)**. **(C)** Representative epitope-specific CD8 T-cell quantification (n=3) before (day 0) and after 7 days of stimulation by distinct pairs of ancestral and mutant SARS-CoV-2 epitopes. ****means p < 0.0001, *means p < 0.

We used an epitope high-throughput screening platform and artificial antigen presentation system to screen HLA-A2-restricted T-cell epitopes and identify all peptides that may contain Omicron mutations (12–14) (**Supplementary Table S1**). The timeline for the overall experimental procedure is shown in **Figure 1A** (14, 15). In total, 42 pairs of predicted epitopes from the variant strains together with the corresponding epitopes from the ancestral strain were synthesized for MHC-I binding and T-cell activation capability screening (**Figure 1B**). We then focused on 3 pairs of epitopes (ORF1a 3673-84/Δ3674-76, S 62-70/A67 V, and S60-71/Δ69-70), with the mutant causing impaired and increased MHC-I binding (**Figures 1C, D**, Z axis), which are located in the ORF1a and spike (S) proteins, respectively. Compared with the corresponding ancestral peptides, the other 39 pairs from the peptide T2 binding assay (Methods) presented decreased MHC-I binding by the variant mutated epitopes (**Supplementary Figures S1A, B**). However, these epitopes could still be constructed as peptide–MHC monomers and further tetramers (**Figure 1D**, Y axis; **Supplementary Figure S1C**). The flow cytometry gating strategy for SARS-CoV-2 epitope-specific CD8^+^ T, GZMB^+^ CD8^+^ T, and IFNγ^+^ CD8^+^ T cells is presented in **Figure 2A**. Tetramer staining revealed a significant increase or reduction in epitope-specific CD8 T cells in the mutated group compared with those in the corresponding ancestral group (**Figures 2B, C**). The representative FACS results of CD8 T cells were selected by tetramers containing the SARS-CoV-2 epitope peptide, and these epitopes did not activate CD8 T cells (**Supplementary Figures S2A, B**, **Supplementary Table S2**). In addition, 1 epitope pair (S 62-70/A67 V) was demonstrated to have an increased CD8 T-cell response after mutation, which also needs further consideration for vaccine design.

### Cytotoxicity of T cells stimulated with T-cell epitopes containing Omicron mutations

**Figure 3:**
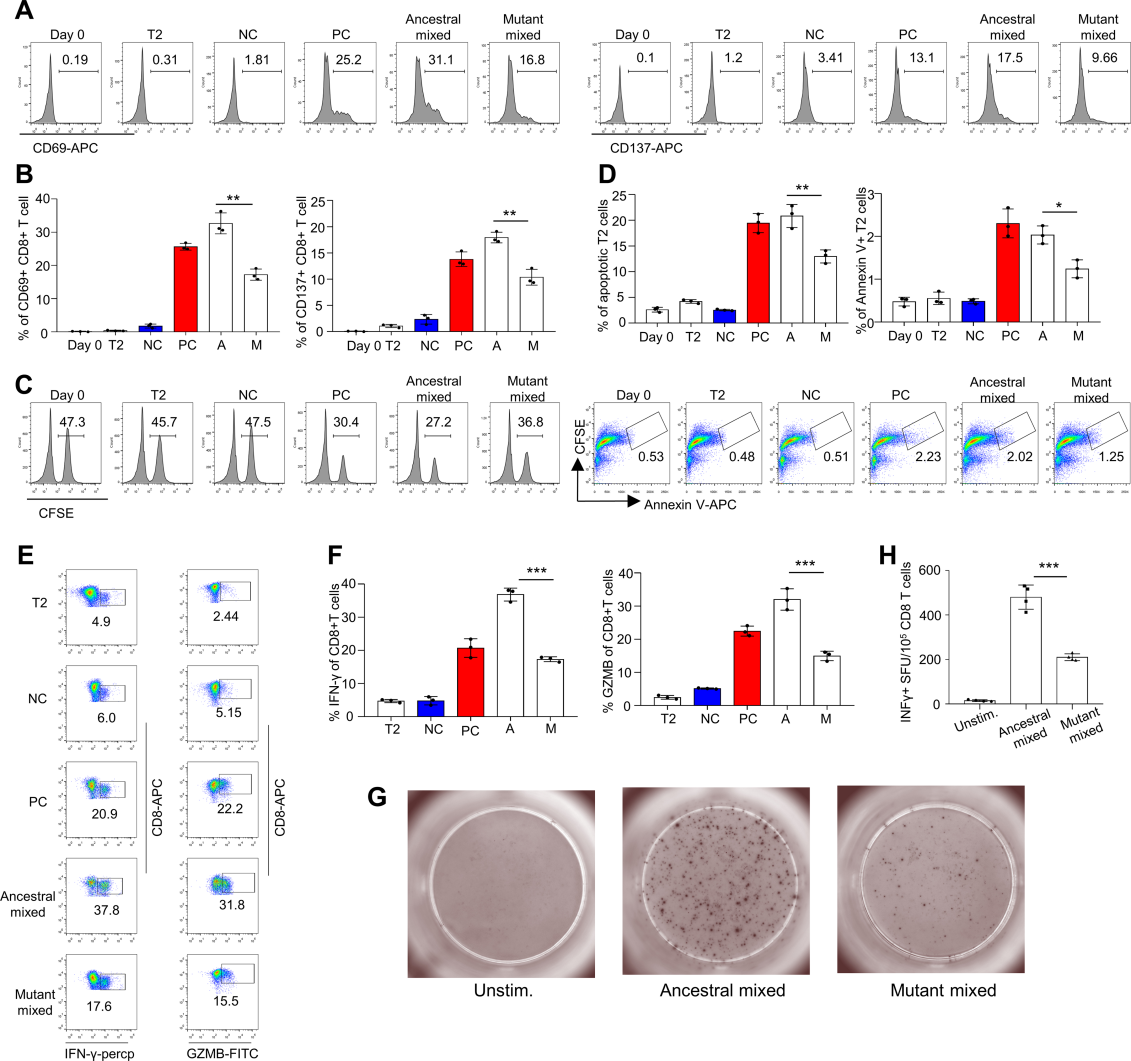
Evaluation of impaired immune protection caused by epitope mutations in the SARS-CoV-2 variant Omicron. **(A, B)** Mitomycin-pretreated T2A2 cells were loaded with mixed peptides from either ancestral or mutant epitopes and incubated with CD8^+^ T cells from healthy donors at a 1:1 ratio. After 16 hours of coculture, the expression of CD69 and CD137 was detected. Representative flow cytometry results and an overall summary of the expression of the CD8 T-cell activation markers CD69 (Left) and CD137 (Right) after cocultivation with T2 cells loaded with a distinct set of peptides (n=3 per experiment). Mixed ancestral or mutant epitopes from the Omicron variant strain were used in each experiment. CD69 and CD137 expression was detected by flow cytometry 16 hours after cocultivation. A: ancestral peptide mixture; M: mutant peptide mixture. **(C, D)** Evaluation of epitope-specific CD8 T-cell-mediated cytotoxicity after 7 days of cell culture. The remaining CFSE-labeled T2 cells were counted and are presented as surviving target cells (left). The values in the flow cytometry chart indicate the percentage of surviving T2 cells. The ratio of CFSE^+^ Annexin V^+^ cells, an indicator of T2 cell apoptosis mediated by epitope-stimulated T cells (right), was calculated. A: ancestral peptide mixture; M: mutant peptide mixture. **(E)** Expression of IFN-γ (left) and granzyme B (right) by CD8^+^ T cells after epitope stimulation for 7 days (n=3). The values in each panel indicate the percentages of IFN-γ^+^CD8^+^ or GZMB^+^CD8^+^ T cells. **(F)** Summary statistics of IFN-γ (left)- and granzyme B (right)-positive CD8^+^ T cells after epitope stimulation for 7 days (n=3). The data shown are the means ± SDs. Each dot represents a single experiment. Statistical significance was determined by a one-tailed t test. **(G, H)** Representative microscopy image **(G)** and summary statistics **(H)** for the anti-IFN-γ ELISpot assay of CD8 T cells stimulated with Omicron ancestral/mutant SARS-CoV-2 epitopes. CD8^+^ T cells (10^5^ per well) from healthy donors were cocultured with T2 cells (10^5^ per well). They were stimulated with ancestral/mutant peptides from Omicron cultured for 4 days in precoated anti-IFN-γ ELISpot plates. n=4 per experiment. Day 0: control before stimulation; T2: T2 control cells without peptide; NC: negative control, T2 cells loaded with the EBV peptide IVTDFSVIK; PC: positive control, T2 cells loaded with the influenza A M1 peptide GILGFVFTL. Consistent control notations are used throughout the paper. ***means p < 0.001, **means p < 0.01, *means p < 0.05.

**Figure 4:**
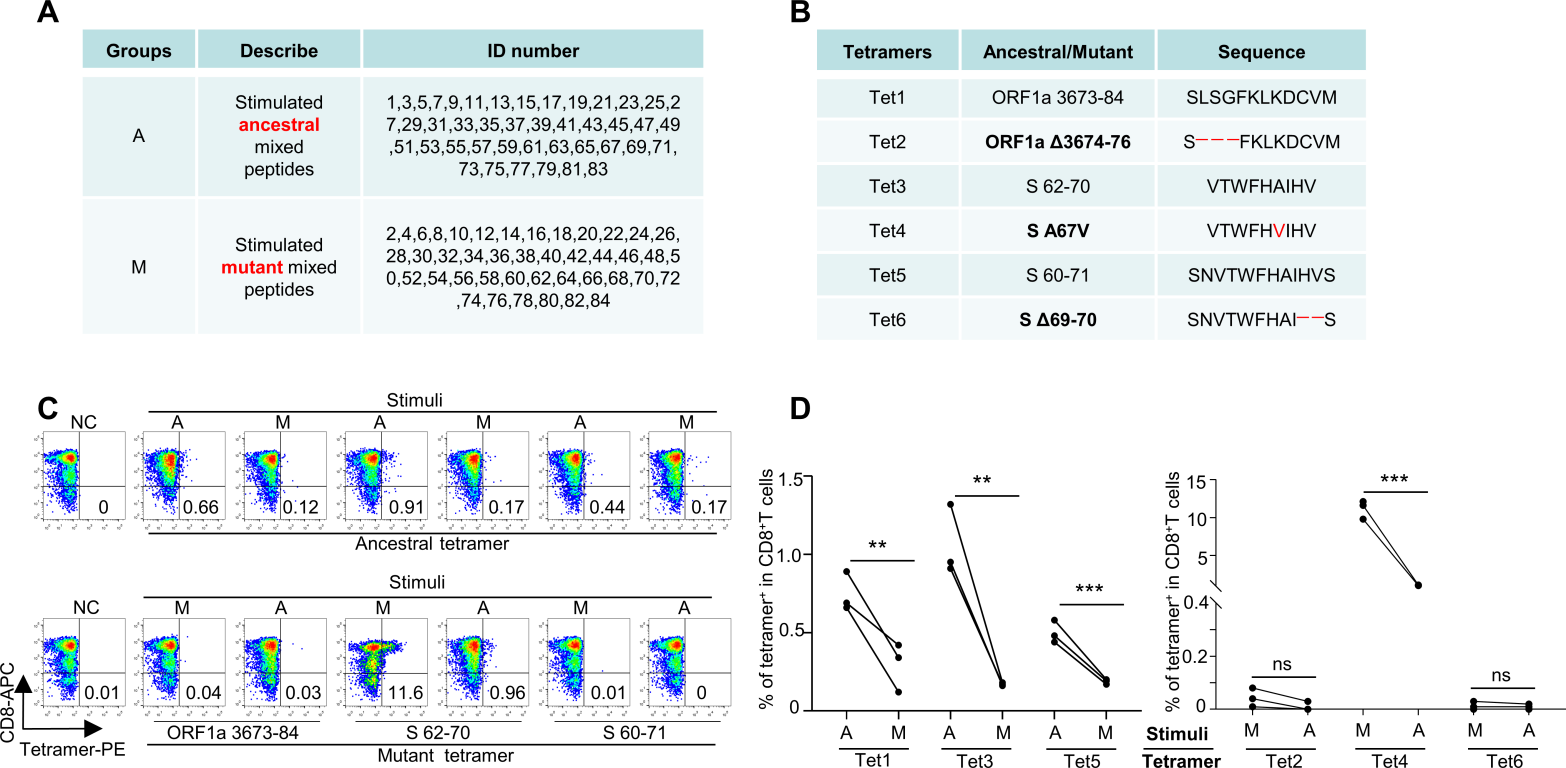
Immune response alteration by SARS-CoV-2 mutant epitopes. **(A)** List of ID number epitopes of ancestral and mutant group stimulus peptides. Group A represents ancestral, whereas Group M represents mutant. The ID numbers correspond to the Omicron variant epitopes listed in **Supplementary Table S1**. **(B)** List of corresponding tetramer epitopes. Tet 1, 3, and 5 represent tetramers of the ancestral epitope, whereas Tet 2, 4, and 6 represent tetramers of the mutant epitope, which are indicated in bold. **(C)** Corresponding epitope-specific CD8 T cells were detected by tetramer cross-reactivity based on ancestral and mutant peptides. Top row: ancestral or mutant epitope-stimulated CD8 T cells stained with ancestral peptide-based tetramers; bottom row: mutant or ancestral epitope-stimulated CD8 T cells stained with mutant peptide-based tetramers. Related to **(A)**. **(D)** Statistics of the cross-detection of epitope-specific CD8 T cells (n=3). Left: ancestral or mutant epitope-stimulated CD8 T cells stained with ancestral peptide-based tetramers; Right: mutant or ancestral epitope-stimulated CD8 T cells stained with mutant peptide-based tetramers. ***means p < 0.001, **means p < 0.01, ns means not statistically significant (p ≥ 0.05).

In the T-cell activation assay using CD8 T cells from healthy HLA-A2^+^ donors (**Supplementary Table S2**), the T cells exhibited reduced activation upon stimulation with the total pool of mutant peptides, as indicated by CD69 and CD137 expression, compared with the ancestral peptides (**Figures 3A, B**). The results of the cytotoxicity assay also revealed impaired cytotoxic ability of CD8 T cells stimulated with mutated epitopes, with decreased killing of target cells (**Figures 3C, D**). The induced IFN-γ and GZMB levels were also lower in the mutant group than in the ancestral group (**Figures 3E, F**). We also analyzed the T-cell responses of healthy donors via ELISpot. CD8 T and T2 cells were stimulated with ancestral/mutant peptides from Omicron and cultured for 4 days in precoated IFN-γ ELISpot plates. The levels of the induced T-cell effector cytokine IFN-γ were lower in the mutant group than in the ancestral group (**Figures 3G, H**; Methods). Next, we performed cross-detection of ancestral peptide-specific CD8^+^ T cells via tetramers containing mutant peptides and vice versa. The ID numbers for the epitopes of the ancestral and mutant groups of stimulus peptides (**Supplementary Table S1**), along with the corresponding tetramer epitopes, are shown in **Figures 4A, B**. The results indicated that CD8^+^ T cells stimulated with mutant peptides could not be recognized by tetramers containing ancestral peptides and that CD8^+^ T cells stimulated with ancestral peptides could not be recognized by tetramers containing mutant peptides (**Figures 4C, D**). This finding suggests that the establishment of new immune responses is required for the mutated epitopes in the given SARS-CoV-2 variants. These results suggest that the mutant epitope is impaired compared with the cellular immune response mediated by ancestral T cells induced by Omicron.

### SARS-CoV-2-specific CD8 T-cell profiling in SARS-CoV-2 vaccines

**Figure 5:**
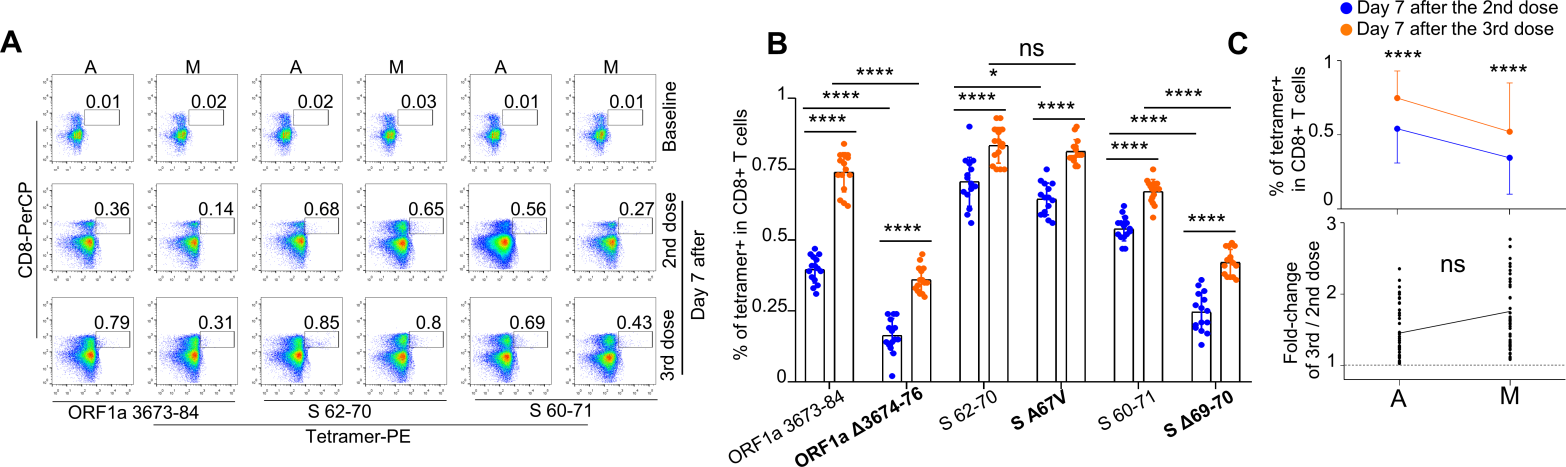
Comparison and characterization of SARS-CoV-2 epitope-specific CD8 T cells between vaccine recipients who received the second and third doses. **(A)** Representative data for the detection of epitope-specific CD8 T cells in HLA-A2^+^ healthy donors before and after the second and third doses (all day 7 after) of the inactivated SARS-CoV-2 vaccine with tetramers prepared with SARS-CoV-2 epitopes. A represents ancestral, whereas M represents mutant. **(B)** Comparison of epitope-specific CD8 T cells between HLA-A2^+^ healthy donors 7 days after the second (blue) and third (orange) doses of the inactivated SARS-CoV-2 vaccine. Specific CD8 T cells were individually stained with tetramers prepared from ancestral and mutant SARS-CoV-2 epitopes. Paired ancestral and mutant epitopes are listed adjacent to each other on the x-axis. The mutant epitope is indicated in bold. n=15 per group. **(C)** Overall statistics and comparison of CD8 T cells specific to ancestral and mutant SARS-CoV-2 epitopes in HLA-A2^+^ healthy donors 7 days after the second (blue) and third (orange) doses (top row). Summary statistics of the detection fold changes in CD8 T cells specific to SARS-CoV-2 epitopes between 7 days after the second and third doses (bottom row). Above the dotted line, the fold change is greater than 1. n=15; the data shown are the means ± SDs. ****means p < 0.0001, *means p < 0.05, ns means not statistically significant (p ≥ 0.05).

We recruited a cohort of 15 HLA-A2^+^ SARS-CoV-2 vaccinees (**Supplementary Table S3**). We then constructed 3 pairs of epitope-based tetramers to examine the production of SARS-CoV-2 antigen-specific CD8 T cells in subjects after inactivated SARS-CoV-2 vaccine administration. On the basis of the results of tetramer staining, SARS-CoV-2 epitope-specific CD8 T cells were detected in all HLA-A2^+^ donors after vaccination. However, the percentage of epitope-specific (ORF1a 3673-84, S 62-70, S 60-71, and corresponding mutation epitopes) CD8 T cells was significantly greater in the third-dose group than in the second-dose group (0.44±0.19% *vs*. 0.64±0.18%, **Figures 5A, B**). Furthermore, after mutation by variant strains, the average number of SARS-CoV-2-specific CD8 T cells decreased (ancestral: 0.54 ± 0.13% *vs*. mutant: 0.34 ± 0.19% at the second dose; ancestral: 0.74 ± 0.08% *vs*. mutant: 0.52 ± 0.19% at the third dose; **Figure 5C** above), indicating potential immune escape of the variant strains (**Figures 5A, C**). However, at the third dose, the ancestral and mutated S protein 67A>V epitopes presented similar proportions of CD8 T specificity (ancestral: 0.83 ± 0.06% *vs*. mutant: 0.82 ±0.04%), indicating that this epitope is a conserved epitope with immunogenicity. When the second and third vaccinations were compared, the percentage of epitope-specific CD8 T cells increased from 0.44% to 0.63% on average, which was a 1.43-fold and 1.75-fold increase for ancestral (ORF1a 3673-84, S 62-70 and S 60-71) and mutant epitopes (ORF1a Δ3674-76, S A67V and S Δ69-70), respectively (**Figure 5C** below). Taken together, the results show that, in addition to the impaired CD8 T-cell response upon Omicron challenge and the efficiency of the third-dose vaccination, the identified epitope S protein 67A>V represents the most conserved sequence of the SARS-CoV-2 variant Omicron and may be used in a variety of vaccine development strategies.

### Cytotoxic function of antigen-specific CD8 T cells induced by the inactivated vaccine

**Figure 6:**
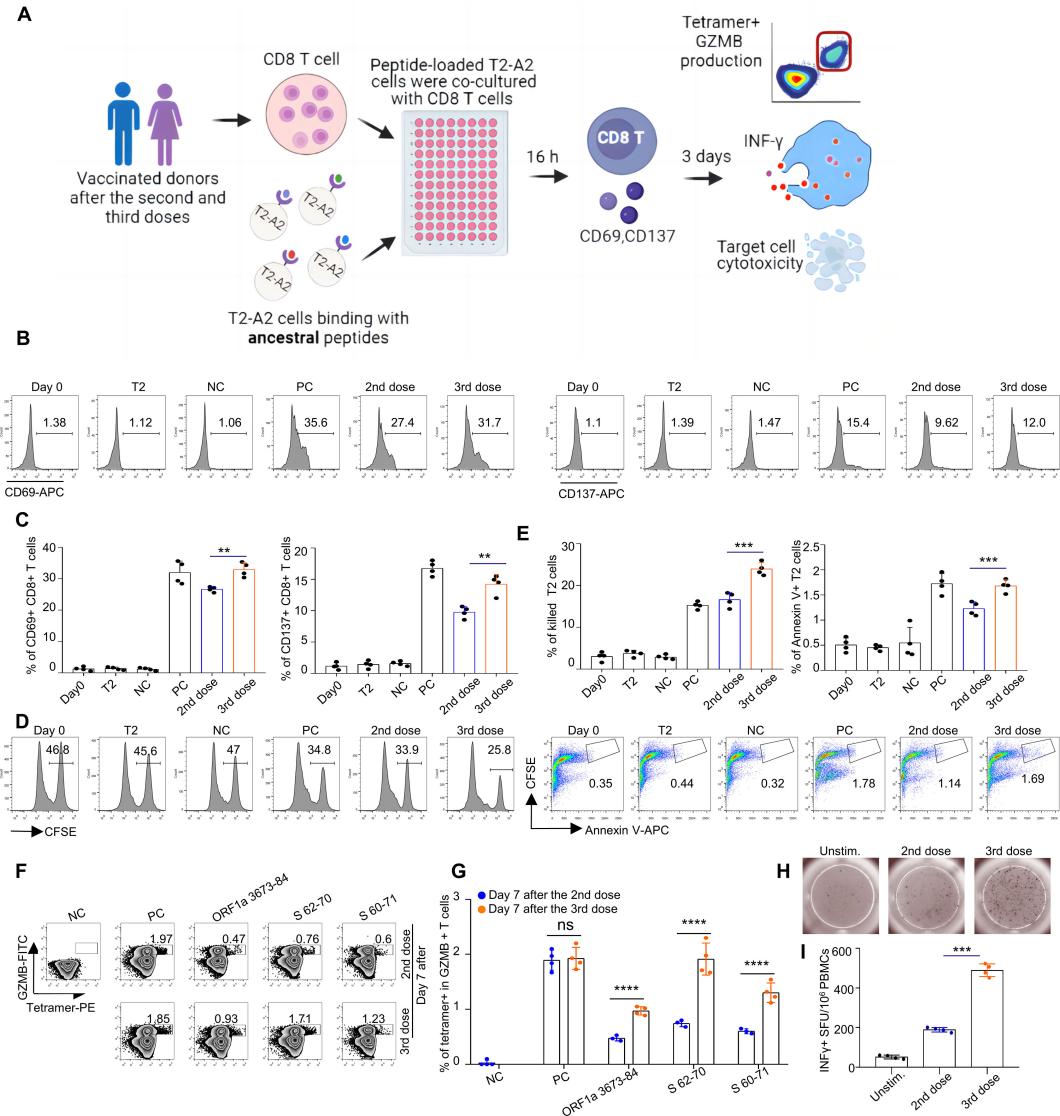
Comparison and characterization of specific CD8+ T-cell-mediated cytotoxic effects between recipients of the second and third vaccination doses. **(A)** General flow chart of the experiment. To compare the efficacy of the second and third doses of inactivated vaccines in HLA-A2 individuals, peripheral blood was collected from vaccinated volunteers 7 days postvaccination. CD8^+^ T cells were isolated from these samples and cocultured with T2A2 cells pulsed with specific peptides. At 16 hours post coculture, the expression of CD69 and CD137 on CD8^+^ T cells was assessed. After 3 days of coculture, antigen-specific CD8^+^ T cells were identified via tetramers, and the expression of IFN-γ and Granzyme B was measured. Additionally, the cytotoxic activity of activated CD8^+^ T cells against target cells was evaluated. **(B-G)** Characterization of epitope-specific CD8^+^ T cells after vaccination. CD8 T cells isolated from vaccinated donors 7 days after the third dose were cocultured with T2 cells loaded with SARS-CoV-2 ancestral epitopes at a 1:1 ratio and analyzed for the expression of CD69 and CD137 after 16 hours **(B, C)**, target cell cytotoxicity **(D, E)** and GZMB production after 3 days **(F, G)**. For the cell cytotoxicity assay, T2 cells were labeled with CFSE and loaded with SARS-CoV-2 epitopes as target cells. Target cell cytotoxicity was assessed by the proportions of killed T2 cells and apoptotic T2 cells. Day 0: control before stimulation; T2: T2 control cells without peptide; NC: negative control, T2 cells loaded with the EBV peptide IVTDFSVIK; PC: positive control, T2 cells loaded with the influenza A M1 peptide GILGFVFTL; 2nd dose: day 7 after the 2nd dose; 3rd dose: day 7 after the 3rd dose. **(B, D, F)** are representative data of **(C,E, G)**, respectively. The data are summarized as the means ± SDs. n=4 for each group. Related to **Supplementary Table S3**, the ID numbers are 1, 7, 9, and 15. **(H, I)** Representative microscopy image **(H)** and summary statistics **(I)** for the anti-IFN-γ ELISpot assay of PBMCs from second- and third-dose vaccinated donors (all day 3 after) stimulated with SARS-CoV-2 ancestral epitopes.

A general flow chart of the experiment is shown in **Figure 6A**. To assess the cytotoxic function of the antigen-specific CD8 T cells induced by the inactivated CoronaVac or BBIBP-CorV vaccine (**Supplementary Table S3**), we stimulated CD8 T cells with artificial antigen-presenting T2 cells loaded with 42 mixed ancestral epitopes. Furthermore, the vaccination-stimulated CD8 T cells in the third dose group presented increased expression levels of CD69 and CD137 (**Figures 6B, C**), increased cytotoxicity to target cells (**Figures 6D, E**), and increased GZMB (**Figures 6F, G**) production compared with those in the second dose group. To further validate this hypothesis, we also analyzed the T-cell responses of the vaccine participants via ELISpot. The results revealed the same cytotoxic effect on vaccine-induced CD8^+^ T cells (**Figures 6H, I**). Our data revealed an overall increase in T-cell responses when immune responses were analyzed after booster vaccination.

## Discussion

Our study aimed to address an important knowledge gap in the potential of the Omicron SARS-CoV-2 variant to evade recognition by CD8^+^ T cells during the human cellular immune response. First, we predicted potential CD8 T-cell epitopes within the ancestral strain of SARS-CoV-2 and studied those within which mutations occurred in the Omicron strain. Forty-two pairs of potential candidate epitopes of the ancestral strain and variant Omicron were obtained. Second, to verify the binding of these predicted epitopes, we examined whether they could be presented on T2 cells, and if the epitopes could bind to HLA-A2, the peptide-MHC complex would be more stable. Our results revealed that most peptides had a greater binding capacity to HLA-A2 than did the negative peptides, whereas most mutant peptides had a slightly lower binding capacity than did the ancestral peptides.

To date, the SARS-CoV-2 mutations that have received the most attention are those in viral spike proteins, including significant mutations in the receptor-binding domain (RBD), N-terminal domain (NTD), and furin cleavage site regions. Some of these mutations directly affect the binding affinity of the ACE2 receptor to the host, which may subsequently alter the viral load, transmissibility, and infectivity (16, 17). Therefore, it is critical to address the extent to which variant mutations affect immunity induced by SARS-CoV-2 variant infection or vaccination. Currently, most studies on immune responses against Omicron focus on humoral immunity. Previous studies have shown that Omicron can escape most existing SARS-CoV-2 antibody drugs targeting the spike region, such as the LY233 CoV016/LY-CoV555 cocktail and REGN-10933/REGN-109876 cocktail (18). However, more studies have shown a significant 6–100-fold increase in neutralizing titers with a 3-dose heterologous or homologous booster vaccine than with a two-dose vaccine (19–21).

To assess cellular immunity against Omicron, a recent study evaluated minimal crossover between mutations with the Omicron variant of SARS-CoV-2 and CD8 T-cell epitopes found in individuals recovering from COVID-19, suggesting that the cellular response to CD8 T-cell early infection must still be effective against the new variant (22). For example, Pardieck et al. demonstrated that a single T-cell epitope-based vaccine conferred robust protection in a murine model of SARS-CoV-2 infection, emphasizing the critical role of T-cell responses in controlling viral replication and disease severity (23). This finding is consistent with that of Prakash et al., who showed that vaccines incorporating highly conserved B cell, CD4^+^, and CD8^+^ T-cell epitopes induced cross-protection against multiple SARS-CoV-2 variants of concern, significantly reducing severe infection, disease, and mortality. These results highlight the importance of T-cell-mediated immunity in combating viral mutations and emerging variants. The significance of T-cell epitopes extends beyond SARS-CoV-2 to include cross-protection against other common cold coronaviruses (e.g., HCoV-OC43 and HCoV-229E) (24). Palatnik-de-Sousa et al. designed a vaccine based on conserved T-cell epitopes from SARS-CoV-2 variants Alpha to Omicron, which elicited broad and durable immune responses. Their work suggested that conserved T-cell epitopes shared among coronaviruses can induce cross-reactive T-cell responses, providing a foundation for pancoronavirus vaccines (25). This cross-reactivity is further supported by Prakash et al, who demonstrated that conserved epitopes could elicit immunity against not only SARS-CoV-2 but also other coronaviruses, potentially mitigating future zoonotic threats (24). Tada et al.reinforced these findings by showing that a single-epitope T-cell-based vaccine protected against SARS-CoV-2 infection in a preclinical model, underscoring the efficacy of T-cell-targeted strategies (26). By targeting conserved regions, T-cell epitopes reduce susceptibility to viral mutations and increase the ability of the vaccine to combat emerging variants.

Our results first revealed that antigen-presenting T2 cells loaded with mixed epitopes could activate T cells from healthy donors. Among them, the proportion of CD8 T cells specific for certain mutant peptides was lower than that of ancestral T cells in the same study subjects. Furthermore, tetramers prepared with mutated epitopes did not efficiently recognize CD8 T cells activated by ancestral peptides, and vice versa. These results suggest that the T-cell-mediated cellular immune responses induced by various Omicron are decreased. Our previous results also revealed L>F mutations in the spike protein epitope FVF *L* VLVPLV, suggesting that the constant evolution of the virus induced immune evasion (12).

In total, 42 pairs of predicted epitopes from the variant strains together with the corresponding epitopes from the ancestral strain were synthesized, covering the ORF1a, ORF1b, S, M, E, and N proteins. The results revealed that 39, 2, and 1 of the mutant epitopes, relative to the ancestral epitopes, presented no, impaired, or increased T-cell activation capability, respectively. The impaired cellular immune response was further confirmed by the detection of epitope-specific CD8 T cells in SARS-CoV-2 vaccine recipients, with the proportion of T cells recognized by the Omicron mutant epitope being lower than that recognized by the ancestral epitope in the same subject. Taken together, our results suggest that the Omicron variant mutates CD8 T-cell epitopes, thereby impairing CD8 T-cell immune responses. Roanne Keeton et al. reported that despite numerous mutations and reduced susceptibility to Omicron neutralizing antibodies, most T-cell responses induced by infection or vaccination cross-recognize this variant (27, 28). The third injection of the inactivated vaccine resulted in an increase in the number of epitope-specific CD8 T cells, which demonstrated that after two doses of the inactivated vaccine as the “priming” shot, a booster dose of a third homologous inactivated vaccine improved cellular immune protection against Omicron. In addition, we identified S protein 67A>V epitopes, which presented similar proportions of CD8 T specificity between the ancestral and mutant strains, indicating that the epitope is a conserved epitope with immunogenicity.

Some “hot spots” are enriched in immunodominant epitopes of T cells in the protein sequence of SARS-CoV-2, which are completely consistent among the existing variants of the new coronavirus. These highly conserved “hot spots” are ideal vaccine targets. In our study, S A67V epitope stimulation elicited much stronger T-cell responses than stimulation with other epitopes did, indicating that the immunogenicity of the S62-70 epitope was enhanced by the mutation. We identified a highly conserved S protein S 62-70/A67 V epitope between Omicron mutants and ancestral strains that can be used as an ideal vaccine target to induce high levels of T-cell immune responses, which may be effective in preventing infection by SARS-CoV-2 Omicron variants. Moreover, compared with S60-71/Δ69-70, the ability to stimulate T cells decreased after mutation of this epitope, and the deleted amino acid position was 69-70, which further illustrates the importance of the entire S62-70 epitope. Vaccine strategies that induce robust memory and effector T-cell responses, together with antibody responses that collectively target conserved antimutation sites, may generate more durable T-cell immunity capable of providing broad protection against possible future mutations.

The incorporation of T-cell components into existing spike-only COVID-19 vaccines offers significant immunological advantages, particularly in enhancing the breadth and durability of protection. The spike protein, while effective in eliciting neutralizing antibodies, is prone to antigenic drift, leading to reduced efficacy against emerging variants. T-cell epitopes, especially those targeting conserved regions of nonspike proteins (e.g., nucleocapsids and membranes), can provide cross-reactive immunity against diverse SARS-CoV-2 variants and even other coronaviruses. Clinical trials, such as the Gritstone study, have demonstrated that epitope-based vaccines incorporating T-cell targets induce robust CD8^+^ and CD4^+^ T-cell responses, which are critical for long-term immunity and viral clearance. These T-cell responses are less susceptible to viral mutations, offering a more resilient defense against evolving strains (29). Additionally, T-cell-mediated immunity can reduce disease severity by targeting infected cells, complementing the antibody-focused protection of spike-only vaccines. This dual approach aligns with the concept of a pancoronavirus vaccine, which addresses both immediate and future pandemic threats. Thus, the integration of T-cell components represents a promising strategy to increase the efficacy and longevity of COVID-19 vaccines. In conclusion, we identified an S A67V cytotoxic lymphocyte (CTL) epitope suitable for evaluating the CD8 T-cell-mediated cellular response and potentially for the development of future COVID-19 vaccine candidates to maximize CTL responses against SARS-CoV-2.

Our current analysis provides useful information for understanding vaccine-elicited SARS-CoV-2-specific CD8^+^ T-cell responses. However, how these escape mechanisms affect overall immunity during viral infection is unclear. To more accurately understand the effects of Omicron on immunity, vaccine efficacy against Omicron variants should be evaluated in real-world studies. In addition, our study focused on HLA-A* 02-restricted epitopes and should include other HLA class I homotypic allotypic restricted CD8 T-cell-specific epitopes to more fully understand SARS-CoV-2-induced immune responses, which should be addressed in future studies.

## Materials and methods

### Human subject enrollment

The institutional review board of Jinan University School of Medicine approved the study (JNUKY-2021-009). Unexposed donors were collected from healthy individuals registered at the Guangzhou Blood Center until September 2019. The donors had no known history of any significant systemic diseases, including but not limited to hepatitis B or C; HIV; diabetes; kidney or liver diseases; malignant tumors; or autoimmune diseases. The samples were confirmed by negative reports of SARS-CoV-2 RNA real-time reverse transcription polymerase chain reaction (RT-PCR) assays (**Supplementary Table S2**). Fifteen healthy HLA-A2 volunteers with no history of COVID-19 infection were enrolled in this study (**Supplementary Table S3**). The participants received the inactivated SARS-CoV-2 vaccine (CoronaVac or BBIBP-CorV) between June 2021 and January 2022. PBMC samples were collected from human blood at baseline (prior to vaccination) and 7 days after the second and third vaccination doses. All the sample information was blinded during all the experiments.

### Isolation of PBMCs

Healthy human whole blood was collected in a heparinized blood vacutainer and kept at room temperature with gentle agitation until processing. PBMCs from healthy individuals were isolated via density gradient centrifugation in a lymphocyte isolation solution. (GE, US). The percent viability was estimated via standard Trypan blue staining. The PBMCs were cryopreserved in fetal bovine serum (LONSERA, Uruguay) supplemented with 10% DMSO (Sigma-Aldrich, US) and stored long-term in liquid nitrogen until use.

### HLA-A2-restricted T-cell epitope prediction

The membrane (M), envelope (E), nucleocapsid (N), ORF, and spike (S) protein sequences of the SARS-CoV-2 ancestral-Hu-1 strain (NC_045512.2) were analyzed via the “MHC I Binding” tool (http://tools.iedb.org/mhci) for CD8 T-cell epitope prediction. The prediction method used was the IEDB website recommendation 2.22 (NetMHCpan EL), and the HLA subtype selected was HLA-A*02:01, which is the most common HLA class I genotype in the Chinese population (30). We performed a similar prediction of epitopes as for Wuhan-Hu-1 with Omicron. The epitope with the best antigen presentation score was used as the candidate peptide of the ancestral strain. Epitopes from mutant strains whose peptide length was >12 aa and whose antigen presentation ability was predicted by VaxiJen 2.0 (http://www.ddg-pharmfac.net/vaxijen/VaxiJen/VaxiJen.html) were excluded. Additionally, epitopes with the same amino acid sequence except for the mutation point were used as candidate peptides for the Omicron variant (**Supplementary Table S1**).

### Peptide screening in T2 cells

The candidate epitopes were synthesized by GenScript Biotechnology Co., Ltd. (Nanjing, China) with a purity >98% and resuspended in sterile water, PBS, or DMSO at 10 mM. The peptide concentration was titrated as described previously (14). T2 cells do not express A2 on the cell surface unless an exogenous peptide is added (31). T2 cells were maintained in 89% IMDM (HyClone, Cat# SH30228, USA) supplemented with 10% FBS (Lonsera, Cat# S711-001S, Uruguay) and 1% penicillin and streptomycin (HyClone, Cat# SV30010, USA). T2 cells were seeded into 96-well plates at 100,000 per well and incubated with peptides at a final concentration of 20 µM for 4 hours at 37°C. DMSO was used as a blank control, the influenza A M1 peptide (M58-66 GILGFVFTL) was used as a positive control, and the EBV virus peptide (IVTDFSVIK) was used as a negative control. The cells were stained with a PE-conjugated anti-human HLA-A2 antibody (BioLegend, Cat# 343305, US) in the dark for 30 min at 4°C and analyzed with a FACS Canto flow cytometer (BD).

### HLA-A2 ELISA

For the specific experimental steps of the HLA-A2 ELISA, please refer to the basis of our previous work (13). The experimental reaction was then stopped with 50 µL of stop solution (2% w/v oxalic acid dihydrate), which was read at 414 nm with an ELISA reader within 30 minutes.

### Generation of antigen-specific HLA-A2 tetramers

A total of 30 µL of peptide exchange monomer (BioLegend, Cat# 280003, US) was mixed in the above step with 3.3 µL of PE streptavidin (BioLegend, Cat# 405203, US) and incubated at 4°C for 30 min in the dark. Finally, 2.4 µL of blocking solution (1.6 µL of 50 mM biotin (Thermo Fisher, Cat# B20656, USA, and 198.4 µL of PBS) was added to stop the reaction, and the mixture was incubated overnight at 4–8°C before use.

### Activation and cytotoxicity analysis of CD8^+^ T cells

T2 cells were loaded with peptides for subsequent CD8^+^ T-cell activation. T2 cells were then treated with 20 µg/mL mitomycin C for 30 min in a 37°C incubator to stop cell proliferation (14) and loaded with the indicated epitope peptides for 4 hours. Peptide-loaded T2 cells were stained with a fluorescent channel of a FITC-conjugated anti-human HLA-A2 antibody (BioLegend, Cat# 343303, US) to analyze the binding efficiency. CD8 T cells were purified from PBMCs via EasySep Human negative selection (Stemcell, Cat# 17953, Canada) with purities greater than 95%. A total of 0.5×10^6^ CD8 T cells isolated from healthy donors were cocultured with 0.5×10^6^ ancestral/mutant peptide (pools of 42 peptides)-loaded T2 cells stained with 5 µmol/L CFSE (TargetMol) and cocultured with 1 µg/mL anti-human CD28 antibodies (BioLegend, Cat# 302901, US) and 50 IU/mL IL-2 (SL PHARM, recombinant human Interleukin-2 (125Ala) injection). The cells were supplemented with 50 IU/mL IL-2 and 20 µM IL-2 mixed with 42 ancestral/mutant peptides every two days throughout the experiment. The CD8 T-cell activation markers CD69 (BioLegend, Cat# 310909, US) and CD137 (BioLegend, Cat# 309809, US) were evaluated after 16 hours. The effects of the apoptosis marker annexin V-APC (BioLegend, Cat# 640919, US) on T2 cells and tetramer-specific CD8 T cells were evaluated 7 days after peptide stimulation intervention. On day 7, the cells were restimulated for 4 hours with mixed peptides in the presence of GolgiPlug (BD, Cat# 550583, US) and 50 IU/mL IL-2, and the production of granzyme B and IFN-γ was examined with PerCP-conjugated anti-human IFN-γ (BioLegend, Cat# 502524, US) and FITC-conjugated anti-human granzyme B (BioLegend, Cat# 515403, US) staining.

### Epitope-specific CD8 T cells were cross-detected

T2 cells were loaded with peptides for subsequent CD8^+^ T-cell activation. T2 cells were then treated with 20 µg/mL mitomycin C for 30 min in a 37°C incubator to prevent cell proliferation. The T2 and CD8 T cells were supplemented with 50 IU/mL IL-2 and 20 µM 42 ancestral/mutant peptides mixed every two days throughout the experiment. The corresponding ancestral epitope was used to label the group of ancestral epitope mixed and mutant epitope mixed stimuli. The ancestral epitope tetramer-labeled ancestral epitope mixed stimulation was used as a control, and the ancestral epitope tetramer-labeled mutant epitope stimulation was used for cross-recognition. The opposite is true for mutant epitope tetramer markers.

### Cell-surface antibodies and tetramer staining

PBMCs were isolated from the peripheral venous blood of healthy individuals vaccinated with the inactivated SARS-CoV-2 vaccine. HLA-A2^+^ healthy donors were identified by flow cytometry without subtype identification. Briefly, 10^6^ PBMCs were stained with a PE-conjugated anti-human HLA-A2 antibody (BioLegend, Cat# 343305, US) and acquired via a flow cytometer for 30 minutes at 4°C in the dark. HLA-A2-positive PBMC samples were further stained with a PE-labeled tetramer (homemade) plus an APC-labeled human CD8 antibody (BioLegend Cat# 344721, US). CD8 T cells were purified from PBMCs via EasySep human negative selection (Stemcell, Cat# 17953, Canada) with a purity greater than 95%. CD8 T cells isolated from vaccinated donors after the second and third doses were cocultured with T2 cells loaded with 42 SARS-CoV-2 ancestral epitopes at a 1:1 ratio. T cells were subjected to PE-labeled tetramer and FITC-conjugated anti-human granzyme B (BioLegend, Cat#515403, US) production after 3 days and acquired with a FACS Canto flow cytometer (BD).

### ELISpot assays

The thawed PBMCs were incubated for 3–4 hours in RPMI 1640 medium supplemented with 10% fetal bovine serum (LONSERA, Uruguay) in a 37°C incubator. The cells were then stimulated with 20 µM peptide pools corresponding to ancestral/mutant peptides from Omicron. The cell suspensions were transferred to a Human IFN-γ Precoated ELISPOT Kit (strips) (Dayou, Cat# 2110005) and developed after 4 days according to the manufacturer’s instructions. The spots were imaged and counted via an ELISpot reader (Mabtech).

### Statistical analysis

The differences in the adaptive immune response, which includes the cellular immune response before the first dose of the inactivated SARS-CoV-2 vaccine and 7 days after the second and third vaccination doses, were analyzed via one-way, two-way repeated-measures ANOVA and paired-samples t tests. All the statistical analyses were performed with GraphPad Prism 8, SPSS 22.0 software and the R statistical package. A *P* value less than 0.05 (two-tailed) was considered statistically significant.

## Data Availability

The original contributions presented in the study are included in the article/**Supplementary Material**. Further inquiries can be directed to Chanchan Xiao, xiaocc616@foxmail.com.

## Supplementary material

Supplementary Figure 1 Assessment of the binding affinity of ancestral and mutated epitopes of the Omicron variant with HLA-A2. **(A, B)** Representative data and summary statistics of ancestral and mutant SARS-CoV-2 epitopes binding affinity to HLA-A2 in T2 cells. Ancestral and mutant epitopes are listed adjacently and in black and bold, respectively. Ancestral: Wuhan strain-derived epitope; Mutant: variant strain-derived epitope. Blank: no peptides; NC: negative control, EBV virus peptide IVTDFSVIK; PC: positive control, influenza A M1 peptide GILGFVFTL. The mutant epitopes were derived from the Omicron strain. **(C)** Evaluation of ancestral and mutant SARS-CoV-2 epitope binding to HLA-A2 by ELISA. The data are shown as the means ± SDs. The threshold for pMHC formation positivity was set as above the average OD value of the negative control. HLA: control UV-sensitive HLA-A2 monomer with UV-sensitive epeptide without UV irradiation. Detailed information on the peptides synthesized from SARS-CoV-2 variants is shown in **Supplementary Table S1**.

Supplementary Figure 2 Characterization of the immunogenicity of the HLA-A2-restricted T-cell epitope. **(A)** Representative FACS results for CD8 T-cell selection via tetramers containing SARS-CoV-2 epitope peptides. CD8+ T cells from healthy donors were cocultured with T2 cells loaded with various peptides for specific CD8+ T-cell recognition. The cells were stained with corresponding tetramers containing ancestral or mutated epitopes after 7 days. The pairs of ancestral and mutated epitopes failed to bind with any CD8 T cells. **(B)** Antigenic value on the X-axis and quantification of activated and nonactivated epitope-specific CD8^+^ T cells on the Y-axis.
