# Supplementary figures and images for: Characterizing HLA-A2-restricted CD8+ T-cell epitopes and immune responses to Omicron variants in SARS-CoV-2-inactivated vaccine recipients
Source: Front Immunol. 2025 Mar 18;16:1534530. doi: 10.3389/fimmu.2025.1534530 (PMC11958996; doi:10.3389/fimmu.2025.1534530)

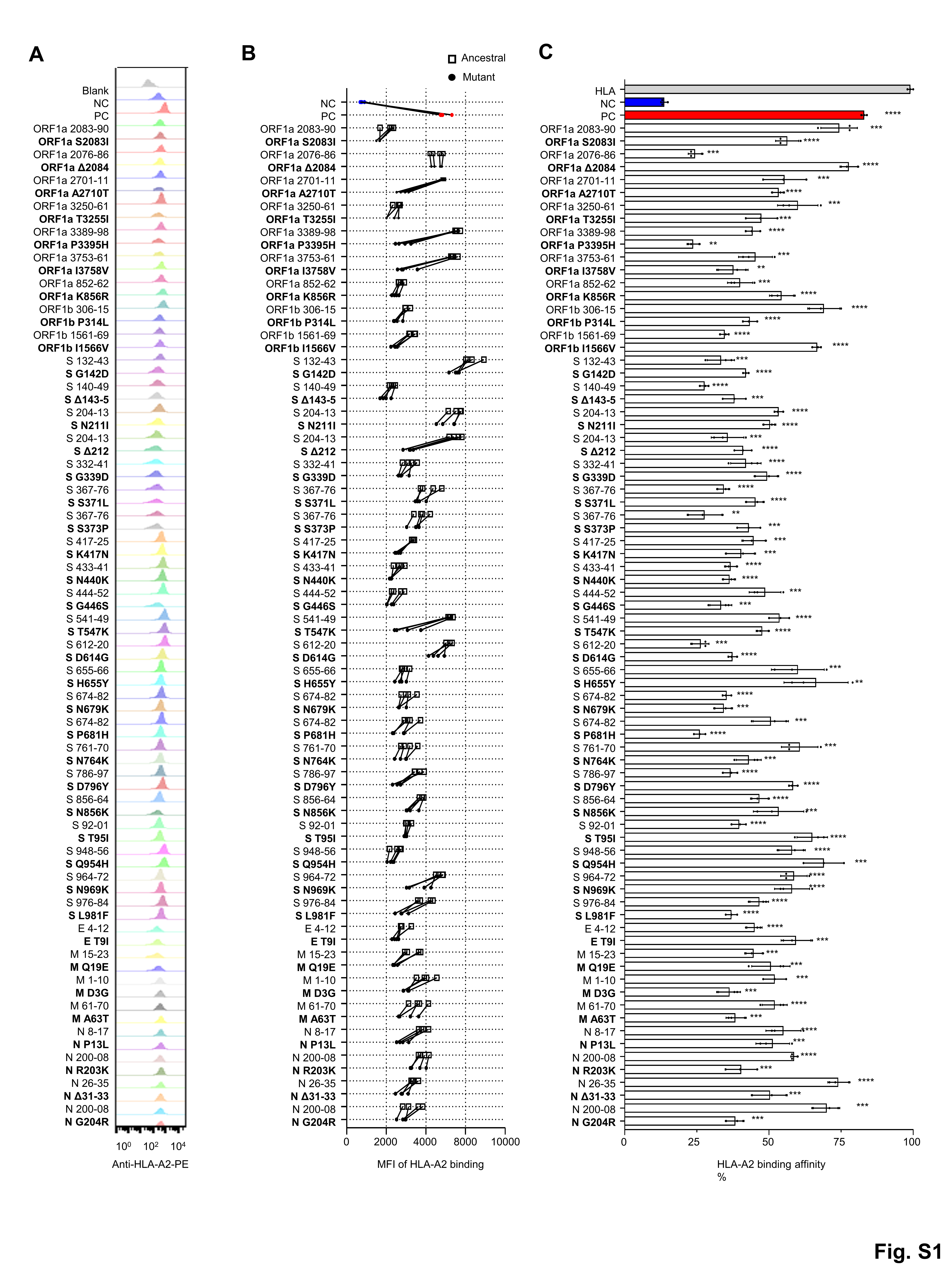

Supplement: Supplementary Figure 1 — Assessment of the binding affinity of ancestral and mutated epitopes of the Omicron variant with HLA-A2. (A, B) Representative data and summary statistics of ancestral and mutant SARS-CoV-2 epitopes binding affinity to HLA-A2 in T2 cells. Ancestral and mutant epitopes are listed adjacently and in black and bold, respectively. Ancestral: Wuhan strain-derived epitope; Mutant: variant strain-derived epitope. Blank: no peptides; NC: negative control, EBV virus peptide IVTDFSVIK; PC: positive control, influenza A M1 peptide GILGFVFTL. The mutant epitopes were derived from the Omicron strain. (C) Evaluation of ancestral and mutant SARS-CoV-2 epitope binding to HLA-A2 by ELISA. The data are shown as the means ± SDs. The threshold for pMHC formation positivity was set as above the average OD value of the negative control. HLA: control UV-sensitive HLA-A2 monomer with UV-sensitive epeptide without UV irradiation. Detailed information on the peptides synthesized from SARS-CoV-2 variants is shown in Supplementary Table S1 . [file Image1.tif]

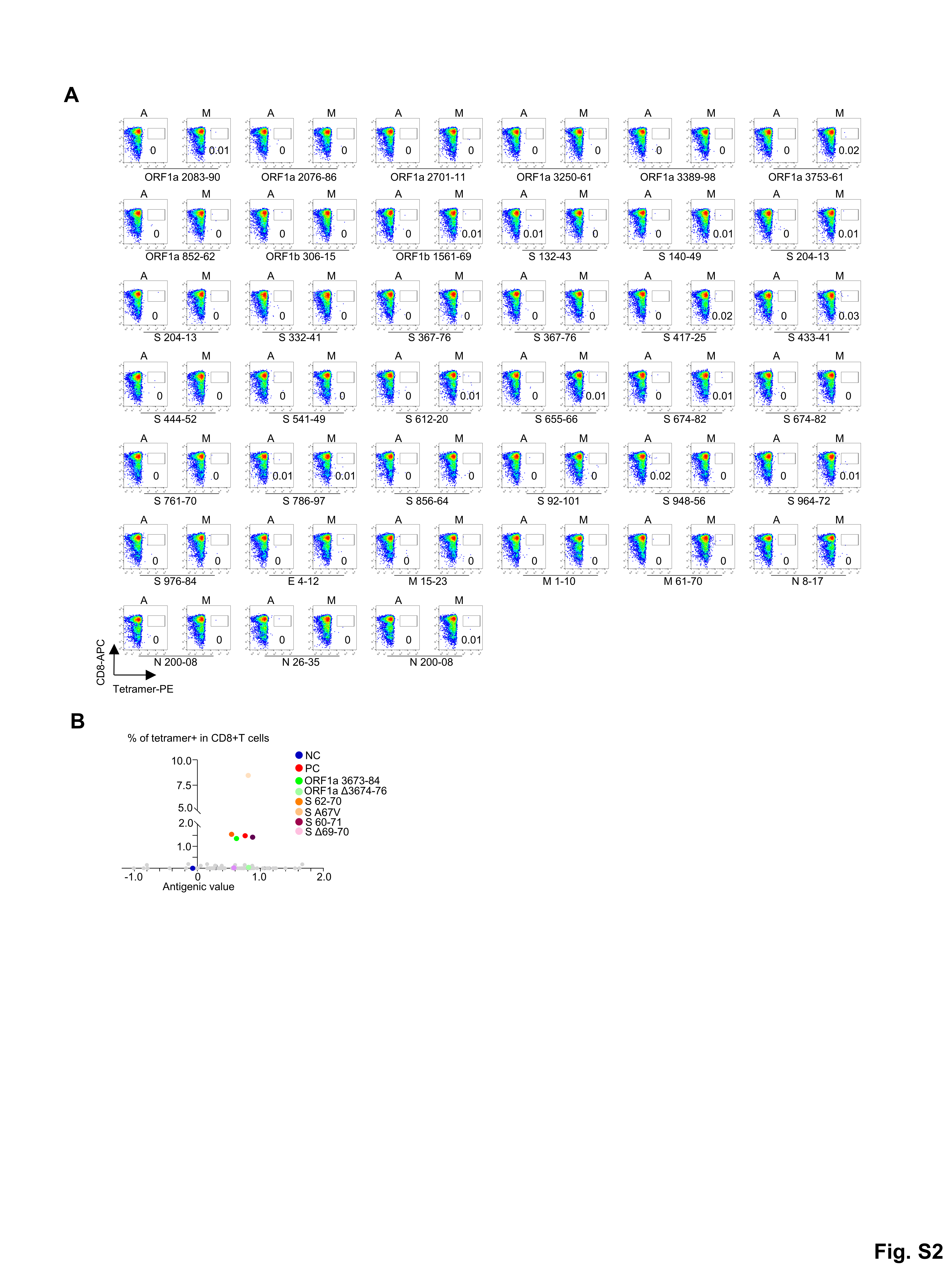

Supplement: Supplementary Figure 2 — Characterization of the immunogenicity of the HLA-A2-restricted T-cell epitope. (A) Representative FACS results for CD8 T-cell selection via tetramers containing SARS-CoV-2 epitope peptides. CD8+ T cells from healthy donors were cocultured with T2 cells loaded with various peptides for specific CD8+ T-cell recognition. The cells were stained with corresponding tetramers containing ancestral or mutated epitopes after 7 days. The pairs of ancestral and mutated epitopes failed to bind with any CD8 T cells. (B) Antigenic value on the X-axis and quantification of activated and nonactivated epitope-specific CD8+ T cells on the Y-axis. [file Image2.tif]
