# Supplementary material for: Characterizing HLA-A2-restricted CD8+ T-cell epitopes and immune responses to Omicron variants in SARS-CoV-2-inactivated vaccine recipients
Source: Front Immunol. 2025 Mar 18;16:1534530. doi: 10.3389/fimmu.2025.1534530 (PMC11958996; doi:10.3389/fimmu.2025.1534530)
Supplement: Supplementary file 3 [file Table1.doc]

Table S1 List of the epitopes of peptides synthesized from SARS-CoV-2 Omicron variants

| **Protein** | **ID** | **Ancestral**  **/mutant** | **Length** | **Start position** | **End**  **position** | **Sequence** | **Antigenic value** |
| --- | --- | --- | --- | --- | --- | --- | --- |
| ORF1a | 1 | Ancestral | 8 | 2083 | 2090 | SLKITEEV | 1.1384 |
|  | 2 | S2083I | 8 | 2083 | 2090 | ILKITEEV | 0.2871 |
|  | 3 | Ancestral | 11 | 2076 | 2086 | ILKPANNSLKI | 0.2931 |
|  | 4 | Δ2084 | 10 | 2076 | 2086 | ILKPANNSKI | 0.2952 |
|  | 5 | Ancestral | 11 | 2701 | 2711 | AQVAKSHNIAL | 0.8342 |
|  | 6 | A2710T | 11 | 2701 | 2711 | AQVAKSHNITL | 0.7347 |
|  | 7 | Ancestral | 12 | 3250 | 3261 | YQPPQTSITSAV | 0.4280 |
|  | 8 | T3255I | 12 | 3250 | 3261 | YQPPQISITSAV | 1.0646 |
|  | 9 | Ancestral | 10 | 3389 | 3398 | YQCAMRPNFT | 0.7906 |
|  | 10 | P3395H | 10 | 3389 | 3398 | YQCAMRHNFT | 0.2931 |
|  | 11 | Ancestral | 12 | 3673 | 3684 | SLSGFKLKDCVM | 0.6197 |
|  | 12 | Δ3674-76 | 9 | 3673 | 3684 | SFKLKDCVM | 0.8119 |
|  | 13 | Ancestral | 9 | 3753 | 3761 | FLARGIVFM | 0.7022 |
|  | 14 | I3758V | 9 | 3753 | 3761 | FLARGVVFM | 0.7039 |
|  | 15 | Ancestral | 11 | 852 | 862 | VLNEKCSAYTV | 0.7507 |
|  | 16 | K856R | 11 | 852 | 862 | VLNERCSAYTV | 0.8023 |
| ORF1b | 17 | Ancestral | 10 | 306 | 315 | VLFSTVFPPT | 0.1585 |
|  | 18 | P314L | 10 | 306 | 315 | VLFSTVFPLT | 0.4331 |
|  | 19 | Ancestral | 9 | 1561 | 1569 | GLCVDIPGI | -0.8017 |
|  | 20 | I1566V | 9 | 1561 | 1569 | GLCVDVPGI | -1.0086 |
| S | 21 | Ancestral | 12 | 132 | 143 | EFQFCNDPFLGV | 0.5987 |
|  | 22 | G142D | 12 | 132 | 143 | EFQFCNDPFLDV | 0.2588 |
|  | 23 | Ancestral | 10 | 140 | 149 | FLGVYYHKNN | 0.8967 |
|  | 24 | Δ143-5 | 7 | 140 | 149 | FLGHKNN | 0.8077 |
|  | 25 | Ancestral | 10 | 204 | 213 | YSKHTPINLV | 1.0391 |
|  | 26 | N211I | 10 | 204 | 213 | YSKHTPIILV | 0.9467 |
|  | 27 | Ancestral | 10 | 204 | 213 | YSKHTPINLV | 1.0391 |
|  | 28 | Δ212 | 9 | 204 | 213 | YSKHTPINV | 0.7381 |
|  | 29 | Ancestral | 10 | 332 | 341 | ITNLCPFGEV | 0.8724 |
|  | 30 | G339D | 10 | 332 | 341 | ITNLCPFDEV | 0.8901 |
|  | 31 | Ancestral | 10 | 367 | 376 | VLYNSASFST | 0.1914 |
|  | 32 | S371L | 10 | 367 | 376 | VLYNLASFST | 0.2069 |
|  | 33 | Ancestral | 10 | 367 | 376 | VLYNSASFST | 0.1914 |
|  | 34 | S373P | 10 | 367 | 376 | VLYNSAPFST | 0.2493 |
|  | 35 | Ancestral | 9 | 417 | 425 | KIADYNYKL | 1.6639 |
|  | 36 | K417N | 9 | 417 | 425 | NIADYNYKL | 1.5485 |
|  | 37 | Ancestral | 9 | 433 | 441 | VIAWNSNNL | 0.7216 |
|  | 38 | N440K | 9 | 433 | 441 | VIAWNSNKL | 0.2326 |
|  | 39 | Ancestral | 9 | 444 | 452 | KVGGNYNYL | 0.5994 |
|  | 40 | G446S | 9 | 444 | 452 | KVSGNYNYL | 0.3915 |
|  | 41 | Ancestral | 9 | 541 | 549 | FNFNGLTGT | 1.2014 |
|  | 42 | T547K | 9 | 541 | 549 | FNFNGLKGT | 1.0434 |
|  | 43 | Ancestral | 9 | 612 | 620 | YQDVNCTEV | 1.6172 |
|  | 44 | D614G | 9 | 612 | 620 | YQGVNCTEV | 1.3957 |
|  | 45 | Ancestral | 12 | 655 | 666 | HVNNSYECDIPI | 0.6752 |
|  | 46 | H655Y | 12 | 655 | 666 | **Y**VNNSYECDIPI | 0.5694 |
|  | 47 | Ancestral | 9 | 62 | 70 | VTWFHAIHV | 0.5426 |
|  | 48 | A67V | 9 | 62 | 70 | VTWFHVIHV | 0.8071 |
|  | 49 | Ancestral | 9 | 674 | 682 | YQTQTNSPR | -0.1787 |
|  | 50 | N679K | 9 | 674 | 682 | YQTQTKSPR | 0.2455 |
|  | 51 | Ancestral | 9 | 674 | 682 | YQTQTNSPR | -0.1787 |
|  | 52 | P681H | 9 | 674 | 682 | YQTQTNSHR | 0.0032 |
|  | 53 | Ancestral | 12 | 60 | 71 | SNVTWFHAIHVS | 0.8768 |
|  | 54 | Δ69-70 | 10 | 60 | 71 | SNVTWFHAIS | 0.5797 |
|  | 55 | Ancestral | 10 | 761 | 770 | TQLNRALTGI | 0.2187 |
|  | 56 | N764K | 10 | 761 | 770 | TQLKRALTGI | 0.3385 |
|  | 57 | Ancestral | 12 | 786 | 797 | KQIYKTPPIKDF | -0.1444 |
|  | 58 | D796Y | 12 | 786 | 797 | KQIYKTPPIKYF | -0.4400 |
|  | 59 | Ancestral | 9 | 856 | 864 | NGLTVLPPL | 0.3547 |
|  | 60 | N856K | 9 | 856 | 864 | KGLTVLPPL | 0.6393 |
|  | 61 | Ancestral | 10 | 92 | 101 | FASTEKSNII | 0.8707 |
|  | 62 | T95I | 10 | 92 | 101 | FASIEKSNII | 0.8524 |
|  | 63 | Ancestral | 9 | 948 | 956 | LQDVVNQNA | 0.0526 |
|  | 64 | Q954H | 9 | 948 | 956 | LQDVVNHNA | -0.0207 |
|  | 65 | Ancestral | 9 | 964 | 972 | KQLSSNFGA | 0.3753 |
|  | 66 | N969K | 9 | 964 | 972 | KQLSSKFGA | 0.2403 |
|  | 67 | Ancestral | 9 | 976 | 984 | VLNDILSRL | -0.8524 |
|  | 68 | L981F | 9 | 976 | 984 | VLNDIFSRL | -0.7889 |
| E | 69 | Ancestral | 9 | 4 | 12 | FVSEETGTL | 0.3864 |
|  | 70 | T9I | 9 | 4 | 12 | FVSEEIGTL | 0.4346 |
| M | 71 | Ancestral | 9 | 15 | 23 | KLLEQWNLV | 0.2095 |
|  | 72 | Q19E | 9 | 15 | 23 | KLLEEWNLV | 0.2339 |
|  | 73 | Ancestral | 10 | 1 | 10 | MADSNGTITV | 0.4240 |
|  | 74 | D3G | 10 | 1 | 10 | MAGSNGTITV | 0.5718 |
|  | 75 | Ancestral | 10 | 61 | 70 | TLACFVLAAV | 1.2318 |
|  | 76 | A63T | 10 | 61 | 70 | TLTCFVLAAV | 1.2460 |
| N | 77 | Ancestral | 10 | 8 | 17 | NQRNAPRITF | 0.8505 |
|  | 78 | P13L | 10 | 8 | 17 | NQRNALRITF | 1.1293 |
|  | 79 | Ancestral | 9 | 200 | 208 | GSSRGTSPA | 0.5500 |
|  | 80 | R203K | 9 | 200 | 208 | GSSKGTSPA | 0.5709 |
|  | 81 | Ancestral | 10 | 26 | 35 | SNQNGERSGA | 0.1546 |
|  | 82 | Δ31-33 | 7 | 26 | 35 | SNQNGGA | 0.2815 |
|  | 83 | Ancestral | 9 | 200 | 208 | GSSRGTSPA | 0.5500 |
|  | 84 | G204R | 9 | 200 | 208 | GSSRRTSPA | 1.1044 |

Δ: amino acid deletion.

The mutated amino acids were highlighted in red.

Table S2 Information on healthy blood sample collection before september 2019

| ID | HLA-A2 | SARS-CoV-2 PCR | Sex | Age |
| --- | --- | --- | --- | --- |
| 1 | Yes | Negative | Female | 34 |
| 2 | Yes | Negative | male | 28 |
| 3 | Yes | Negative | male | 28 |
| 4 | Yes | Negative | Female | 35 |
| 5 | No | Negative | male | 31 |
| 6 | No | Negative | Female | 29 |
| 7 | No | Negative | male | 35 |
| 8 | No | Negative | male | 32 |
| 9 | No | Negative | Female | 36 |
| 10 | No | Negative | Female | 26 |

Table S3 Participants information of inactivated SARS-CoV-2 vaccine cohort

| ID | HLA-A2 | Sex | Age | BMI  (kg/m^2^) | Vaccine |
| --- | --- | --- | --- | --- | --- |
| 1^#^ | Yes | Female | 24 | 22.1 | CoronaVac |
| 2 | Yes | Female | 20 | 21.1 | CoronaVac |
| 3 | Yes | Female | 23 | 22.0 | CoronaVac |
| 4 | Yes | Female | 25 | 20.2 | CoronaVac |
| 5 | Yes | Female | 21 | 22.9 | CoronaVac |
| 6 | Yes | Female | 20 | 17.9 | CoronaVac |
| 7^#^ | Yes | Male | 20 | 20.2 | CoronaVac |
| 8 | Yes | Female | 20 | 18.4 | CoronaVac |
| 9^#^ | Yes | Female | 19 | 21.0 | CoronaVac |
| 10 | Yes | Male | 21 | 20.8 | CoronaVac |
| 11 | Yes | Male | 20 | 18.3 | BBIBP-CorV |
| 12 | Yes | Female | 20 | 23.8 | CoronaVac |
| 13 | Yes | Female | 20 | 18.7 | CoronaVac |
| 14 | Yes | Male | 20 | 25.9 | BBIBP-CorV |
| 15^#^ | Yes | Male | 24 | 23.2 | CoronaVac |

#：Comparison and characterization of SARS-CoV-2 epitope-specific CD8 T cells between vaccine recipients who received the second and third doses，Related to Fig. 6A-I.
